# Sex-Specific Differences in the Association Between Race/Ethnicity and NAFLD Among US Population

**DOI:** 10.3389/fmed.2021.795421

**Published:** 2021-12-02

**Authors:** Magda Shaheen, Katrina M. Schrode, Deyu Pan, Dulcie Kermah, Vishwajeet Puri, Ali Zarrinpar, David Elisha, Sonia M. Najjar, Theodore C. Friedman

**Affiliations:** ^1^College of Medicine, Charles R Drew University, Los Angeles, CA, United States; ^2^Heritage College of Osteopathic Medicine, Ohio University, Athens, OH, United States; ^3^University of Florida College of Medicine, Gainesville, FL, United States

**Keywords:** sex, non-alcoholic fatty liver disease (NAFLD), NHANES 2017–2018, race/ethnicity, disparity

## Abstract

Non-alcoholic fatty liver disease (NAFLD) is spreading worldwide, with a racial/ethnic disparity. We examined the gender role in the racial/ethnic difference in NAFLD in the US population. We analyzed data for 3,292 individuals ≥18 years old from NHANES 2017–2018, a representative sample of the non-institutionalized adult population in the US. Exclusions were subjects with elevated transferrin level, chronic hepatitis B or C, excessive alcohol use, or prescription medications that might cause hepatic steatosis. NAFLD was diagnosed by FibroScan^®^ using controlled attenuation parameter (CAP) values: S0 <238, S1 = 238–259, S2 = 260–290, S3 >290. Data were analyzed using Chi square and multinomial regression. The overall prevalence of NAFLD was 47.9% [S2 = 16.1%, and S3 = 31.8%]. The prevalence of S3 was highest among Mexican Americans (46%), lowest among Blacks (22.7%), 29.9% in other Hispanics and 32.1% in Whites (*p* < 0.05). It was higher among Mexican American males (54.1%) compared to Mexican American females (37.7%) (*p* < 0.05). In the adjusted model, Mexican Americans were two times more likely than Whites to have S2 and S3 (*p* < 0.05). Only male Mexican Americans had higher odds of S2 and S3 relative to male White (*p* < 0.05). Males had higher odds of S3 relative to non-menopausal females (*p* < 0.05). There was no difference in the odds of S2 or S3 NAFLD among the menopausal females with or without hormone therapy relative to non-menopausal females (*p* > 0.05). While Mexican Americans had the highest prevalence of severe NAFLD relative to the other racial/ethnic groups, only male Mexican Americans, but not females, had higher likelihood of both moderate and severe NAFLD relative to Whites. Interventions that specifically target Mexican American males are needed to increase awareness about NAFLD and its prevention.

## Introduction

Non-alcoholic fatty liver disease (NAFLD) is a public health concern affecting about 100 million Americans (1), and the prevalence of NAFLD has been increasing (2). In the United States, the prevalence of NAFLD has risen from 18% in 1988–1991 to 31% in 2011–2012 (3). NAFLD is the most common form of liver disease and is the second leading reason for liver transplants in the United States (4, 5). Due to the impact of NAFLD on the health of individuals, in the United States, the health care cost for NAFLD is estimated to be $103 billion (6). There is a range of severity associated with NAFLD. The spectrum ranges from hepatic steatosis, to non-alcoholic steatohepatitis (NASH), fibrosis, and cirrhosis (7). NASH is a progressive form of NAFLD and research shows that it could lead to fatal conditions including cirrhosis and hepatocellular carcinoma (8–10). Among individuals classified as having severe obesity, one out of four have NASH (11). Risk factors for developing NAFLD include mainly age, race/ethnicity and metabolic conditions, and NAFLD is also known to be associated with an increased risk with obesity, type 2 diabetes, metabolic syndrome, and cardiovascular diseases (12–14).

Previous studies have shown that there is a racial/ethnic disparity with NAFLD (1). Hispanics are known to have a higher prevalence of NAFLD and blacks a lower prevalence of NAFLD compared to non-Hispanic Whites (2, 15). We recently found that Mexican Americans were about 2 times more likely than non-Hispanic Whites to have advanced hepatic steatosis, while other Hispanics showed no difference from Whites (16). In patients with biopsy-proven NAFLD and severe obesity, studies show that Whites were significantly more likely to have NAFLD, NASH and advanced fibrosis compared to blacks (17). The risk for NASH among patients with NAFLD was higher in Hispanics and lower in Blacks. In addition, studies have shown genetic factors contribute to the racial/ethnic differences in NAFLD (18–21). Although it has been shown that Whites are more likely to be re-admitted for cirrhosis, an analysis of the Nationwide Inpatient Sample showed that Blacks and Hispanic were less likely to receive liver transplants compared to Whites, and blacks had a higher in-hospital mortality than Whites (22). Moreover, whereas fibrosis has been linked with NAFLD, over time patients remain stable; hence, there is an opportunity to intervene during this time (23, 24).

There have been conflicting results regarding the role of gender as a risk factor for NAFLD, with most population-based studies finding a higher prevalence of NAFLD among males (25), although patients with biopsy-proven NASH in the NASH Clinical Research Network are more likely to be females (26). In one of few studies examining both gender and race in NAFLD, Browning et al. (27) found that sex differences existed only among Whites, and the presence of a sex difference also varied between Hispanics of different heritage (28).

The current study aimed to examine the role of gender and menopausal status in the association between racial/ethnic disparity and NAFLD. We hypothesized that there is a gender disparity in NAFLD among American Hispanic population.

## Research Design and Methods

### Study Population

We analyzed data for 3,292 participants 18 years and older using the National Health and Nutrition Examination Survey (NHANES) 2017–2018. NHANES samples the US population using a complex, multistage probability design, and obtains informed consent from all participants. NHANES protocols were approved by the National Center for Health Statistics Research Ethics Review Board. Our analysis of these publicly-available data was exempt from Charles R. Drew University IRB review.

### Dependent Variable

Liver fibrosis was measured by FibroScan^®^ which uses ultrasound and vibration controlled transient elastography (VCTETM) to derive liver stiffness. The device also simultaneously measures the ultrasound attenuation related to the presence of hepatic steatosis and records the controlled attenuation parameter (CAPTM) as the indicator for liver fat. We categorized the steatosis status using the median CAP dB/m for steatosis grades whereby S0 (no steatosis) <238; S1 (mild steatosis) = 238–259; S2 (moderate steatosis) = 260–290; and S3 (severe steatosis) >290. Subjects were considered to have NAFLD if they had hepatic steatosis and did not have any exclusion criteria. Exclusion criteria included elevated transferrin level >50%, chronic hepatitis B, chronic hepatitis C, excessive alcohol use, or prescription medications that might cause hepatic steatosis. Chronic hepatitis B was defined as positive results for both the hepatitis B surface antigen and hepatitis B core antibody tests. Chronic hepatitis C was defined as positive results for both the hepatitis C antibody and RNA tests. Excessive alcohol use was defined as an average of more than 2 drinks/day for men or 1 drink/day for women. Average alcohol use was determined using the responses to the two questions: “how often drank any type of alcoholic beverage” and “average drinks on a day when drank alcohol” to calculate a daily average. We excluded subjects if they were taking any of the following hepatotoxic drugs: corticosteroids, antiarrhythmics, anticancer-antimetabolites, anticancer-hormonal drugs, anti-convulsant drugs, or nucleoside/nucleotide reverse transcriptase inhibitors.

### Independent Variables and Measures

In our analyses, we included demographic variables (age, gender, race/ethnicity, education, language spoken, and poverty), menopausal status for women, physical activity status, smoking status, diet quality (healthy eating index), body composition (waist-to-hip ratio and body mass index), and laboratory values [cholesterol, HDL, triglyceride, glucose, hemoglobin A1c (HbA1c), highly-sensitive C-reactive protein (hsCRP), AST, and ALT].

Physical activity was based on activity during work, commuting, or leisure time and categorized into 3 categories based on national guidelines which recommend moderate exercise 5 or more times/week or vigorous exercise 3 times/week [0 = inactive (no activity); 1 = does not meet guidelines; and 2 = meets guidelines]. Age was categorized as 18–19 years; 20–34 years; 35–49 years; 50–64 years, and ≥65. Education was categorized as less than high school (<12 grade), high school (12 grade), some college, and at least college degree. Gender was categorized as male and female. Females were further categorized by menopausal status. They were considered menopausal if they stated they had not had “at least one period in the last 12 months,” with menopause given as the reason. The menopausal group was further categorized into those taking or not taking hormone replacements based a yes/no response to the question “Have you ever used female hormones such as estrogen and progesterone? Please include any forms of female hormones, such as pills, cream, patch, and injectable, but do not include birth control methods or use for infertility.” Race/ethnicity was categorized as non-Hispanic White, non-Hispanic Black, Mexican American, Other Hispanics, and Other race, including multi-racial. Language spoken at home was classified as English, Spanish, both, and other languages. Federal income ratio (FIR) was classified as <1, 1–2, and >2 times the federal poverty level. Smoking status was categorized into non-smoker, former, and current smoker. Participants were classified using body mass index (BMI) with BMI <25 (normal), BMI = 25–29.9 (overweight), and BMI = 30 and higher (obese). Waist-to-hip ratio was classified as risk for women (≥0.85)/risk for men (≥1.0) vs. healthy. Diet quality using the healthy eating index score was categorized as good quality diet, needs improvement in quality diet, and poor-quality diet. Based on the hemoglobin A1c (HbA1c), subjects were classified as normal (healthy) (<5.7%), with pre-diabetes (5.7–6.4%), and with diabetes (6.5% and higher). Total cholesterol was categorized as normal (<200 mg/dL), elevated (200–239 mg/dL), and high (≥240 mg/dL). High-density lipoprotein (HDL) was categorized as low (<40 mg/dL), borderline (40–59 mg/dL), and healthy (≥60 mg/dL). Triglyceride level was categorized as normal (<150 mg/dL), borderline (150–199 mg/dL), and high (≥200 mg/dL). High sensitivity C-reactive protein (hsCRP) was categorized as normal (0.1–1.0 mg/dL), mild inflammation (1.0–3 mg/dL), significant inflammation (3–10 mg/dL), and highly significant inflammation (≥10 mg/dL). Alanine aminotransferase (ALT) was categorized as normal (<56 U/L) and elevated (≥56 U/L); and aspartate aminotransferase (AST) was categorized as normal (<40 U/L) and elevated (≥40 U/L).

### Statistical Analyses

We used descriptive statistics including unweighted number and weighted percent for categorical variables. Missing data was 12.75% for FIR and <8% for all other variables. Bivariate analysis using Chi-Square test for categorical variables were used to determine the statistical difference between the racial/ethnic groups and the other independent variables in the prevalence of NAFLD. We performed multinomial regression analysis with listwise deletion to determine the racial/ethnic difference as well as the associated factors of NAFLD stages adjusting for the confounding variables. In order to examine the role of gender in the relationship between race/ethnicity and NAFLD, we conducted a stratified analysis multinomial regression analysis for males and females. The data are presented as adjusted odds ratio and 95% confidence interval. *P*-value of <0.05 was considered statistically significant. The data were analyzed using SAS (Release V.9. 3, 2002; SAS, Inc.). We used the sample weights provided by the NCHS to correct for differential selection probabilities and to adjust for non-coverage and non-response. All estimates were weighted as supplied by NHANES, and the design is being taken into consideration.

## Results

### Population Characteristics

Of the 3,292 subjects in our sample from NHANES 2017–2018, 27.6% were 50–64 years of age and 18.2% were 65 years and older; 9.9% were Black, 8.5% were Mexican Americans, and 6% were other Hispanics. About half of the population were male (48.2%), 9.9% had less than high-school education, and 12.4% were poor (FIR <1). Most of the participants (82.9%) spoke English at home, 15.6% were current smokers, 18.5% were physically-inactive (did no exercise), 71.4% had poor diet, 53.7% had high waist-to-hip ratio (≥0.85 for males, ≥0.9 for females) and 41% were obese by BMI. 10.2% had high total cholesterol (≥240 mg/dL), 15.4% had low HDL, 16.6% had high level of triglyceride (≥200 mg/dL), and 32.6% had significant inflammation as indicated by >3 mg/dL hsCRP level, 3.9% had abnormal AST, 3.6% had abnormal ALT, 23.8% had pre-diabetes, and 9% had diabetes (Table 1).

**Table 1:** Population characteristics by NAFLD status, NHANES 2017–2018.

	**Overall**	**NAFLD**			
		**No/mild (<260)**	**Moderate (260–290)**	**Severe (>290)**	***p*-value**
**Overall**	3,292	1,668 (52.1%)	539 (16.1%)	1,085 (31.8%)	
**Race/ethnicity**					<0.0001
Mexican American	441 (8.5)	157 (36.9)	75 (17.2)	209 (46.0)	
Other Hispanic	286 (6.0)	147 (56.4)	44 (13.8)	95 (29.9)	
Non-Hispanic White	1,220 (65.6)	605 (52.4)	194 (15.5)	421 (32.1)	
Non-Hispanic Black	718 (9.9)	424 (61.2)	115 (16.1)	179 (22.7)	
Other race	627 (9.9)	335 (51.6)	111 (20.6)	181 (27.8)	
**Age (years)**					<0.0001
18–19	165 (3.3)	130 (83.7)	10 (5.5)	25 (10.8)	
20–34	742 (27.4)	479 (65.8)	97 (11.5)	166 (22.7)	
35–49	728 (23.6)	352 (51.1)	124 (17.4)	252 (31.5)	
50–64	922 (27.6)	377 (42.1)	173 (18.7)	372 (39.2)	
65+	735 (18.2)	330 (42.4)	135 (19.3)	270 (38.4)	
**Sex**					0.0016
Male	1,596 (48.2)	745 (47.5)	254 (16.0)	597 (36.5)	
Female	1,696 (51.8)	923 (56.4)	285 (16.2)	488 (27.4)	
**Education**					0.0036
Less than high school	576 (9.9)	286 (50.5)	95 (19.7)	195 (29.9)	
High school	821 (28.1)	421 (50.6)	120 (13.9)	280 (35.5)	
Some college	1,097 (30.9)	536 (49.2)	180 (15.1)	381 (35.7)	
At least college degree	798 (31.1)	425 (57.0)	144 (17.9)	229 (25.1)	
**Language spoken at home**					0.1064
English	2,358 (82.9)	1,244 (53.2)	376 (16.0)	738 (30.8)	
Spanish	219 (3.5)	83 (39.2)	40 (17.4)	96 (43.4)	
Both	357 (7.4)	155 (47.5)	53 (14.2)	149 (38.3)	
Other	358 (6.2)	186 (50.5)	70 (18.4)	102 (31.0)	
**Federal income ratio (FIR)**					0.3495
<1	616 (12.4)	345 (57.4)	80 (13.2)	191 (29.4)	
1–2	890 (19.6)	435 (51.7)	155 (18.0)	300 (30.3)	
>2	1,786 (68.0)	888 (51.3)	304 (16.1)	594 (32.6)	
**Waist-hip ratio**					<0.0001
Healthy	1,442 (46.3)	966 (70.3)	203 (12.1)	273 (17.6)	
Risk for women (≥0.85)/risk for men (≥1.0)	1,850 (53.7)	702 (36.5)	336 (19.5)	812 (44.0)	
**Body mass index (BMI)**					<0.0001
Normal or healthy (<25)	934 (28.0)	793 (88.7)	83 (7.0)	58 (4.3)	
Overweight (25- <30)	1,051 (31.0)	544 (54.1)	208 (20.7)	299 (25.3)	
Obese (≥30)	1,307 (41.0)	331 (25.7)	248 (18.9)	728 (55.5)	
**Smoking status**					<0.0001
Current	550 (15.6)	333 (60.2)	70 (14.9)	147 (24.9)	
Former	758 (24.3)	302 (41.6)	137 (18.8)	319 (39.6)	
Non-smoker	1,984 (60.0)	1,033 (54.3)	332 (15.3)	619 (30.4)	
**Alcohol use**					0.1039
Current drinker	2,248 (76.3)	1,159 (53.3)	372 (16.2)	717 (30.5)	
Former drinker	642 (16.1)	295 (47.5)	109 (18.9)	238 (33.6)	
Never drank	339 (7.6)	180 (50.7)	50 (10.8)	109 (38.5)	
**Physical activity**					<0.0001
Inactive	733 (18.5)	318 (43.9)	133 (17.6)	282 (38.5)	
Does not meet guideline	515 (15.2)	241 (43.2)	77 (14.6)	197 (42.2)	
Meets guidelines	2,044 (66.2)	1,109 (56.5)	329 (16.0)	606 (27.5)	
**Healthy eating index**					0.0110
Poor diet	2,304 (71.4)	1,157 (49.8)	379 (16.8)	768 (33.5)	
Needs improvement	887 (26.0)	460 (56.8)	140 (14.7)	287 (28.5)	
Good diet	101 (2.6)	51 (70.0)	20 (10.9)	30 (19.1)	
**Serum cholesterol**					0.0112
Good (<200 mg/dL)	2,137 (62.7)	1,143 (55.2)	330 (15.7)	664 (29.1)	
Elevated (200–239 mg/dL)	825 (27.1)	384 (47.7)	147 (16.4)	294 (35.9)	
High (≥240 mg/dL)	330 (10.2)	141 (45.0)	62 (17.7)	127 (37.4)	
**High-density lipoproteins**					<0.0001
Low (<40 mg/dL)	556 (15.4)	157 (28.4)	91 (15.3)	308 (56.3)	
Borderline risk (40–59 mg/dL)	1,785 (54.5)	853 (48.3)	301 (16.9)	631 (34.8)	
Healthy (≥60 mg/dL)	951 (30.1)	658 (71.2)	147 (15.0)	146 (13.7)	
**Serum triglycerides**					<0.0001
Normal (<150 mg/dL)	2,236 (67.5)	1,370 (63.6)	339 (14.9)	527 (21.5)	
Borderline (150–199 mg/dL)	505 (15.9)	165 (34.1)	113 (22.6)	227 (43.2)	
High (≥200 mg/dL)	551 (16.6)	133 (22.8)	87 (14.6)	331 (62.5)	
**High-Sensitivity CRP**					
Normal (0.1– <1 mg/dL)	1,004 (31.2)	705 (71.9)	137 (12.4)	162 (15.6)	<0.0001
Mild inflammation (1– <3 mg/dL)	1,171 (36.2)	559 (49.1)	217 (19.7)	395 (31.2)	
Significant inflammation (3– <10 mg/dL)	884 (25.9)	328 (37.7)	144 (15.6)	412 (46.7)	
High significant inflammation (≥10 mg/dL)	233 (6.7)	76 (31.9)	41 (15.7)	116 (52.4)	0.0367
**Aspartate aminotransferase (AST)**					
Normal (≤ 40 U/L)	3,165 (96.1)	1,617 (52.6)	524 (16.2)	1,024 (31.1)	
Elevated (>40 U/L)	127 (3.9)	51 (39.4)	15 (12.5)	61 (48.1)	
**Alanine aminotransferase (ALT)**					0.0031
Normal (≤ 56 U/L)	3,179 (96.4)	1,634 (52.8)	526 (16.2)	1,019 (31.0)	
Elevated (>56 U/L)	113 (3.6)	34 (33.2)	13 (13.0)	66 (53.7)	
**Hemoglobin A1c (HbA1c)**					<0.0001
Healthy (<5.7%)	1,902 (67.2)	1,194 (62.5)	287 (15.8)	421 (21.7)	
Pre-diabetes (5.7–6.4%)	953 (23.8)	378 (36.4)	185 (18.0)	390 (45.5)	
Diabetes (≥6.5%)	437 (9.0)	96 (16.2)	67 (13.1)	274 (70.6)	

### Prevalence of NAFLD Stages

**Figure 1:**
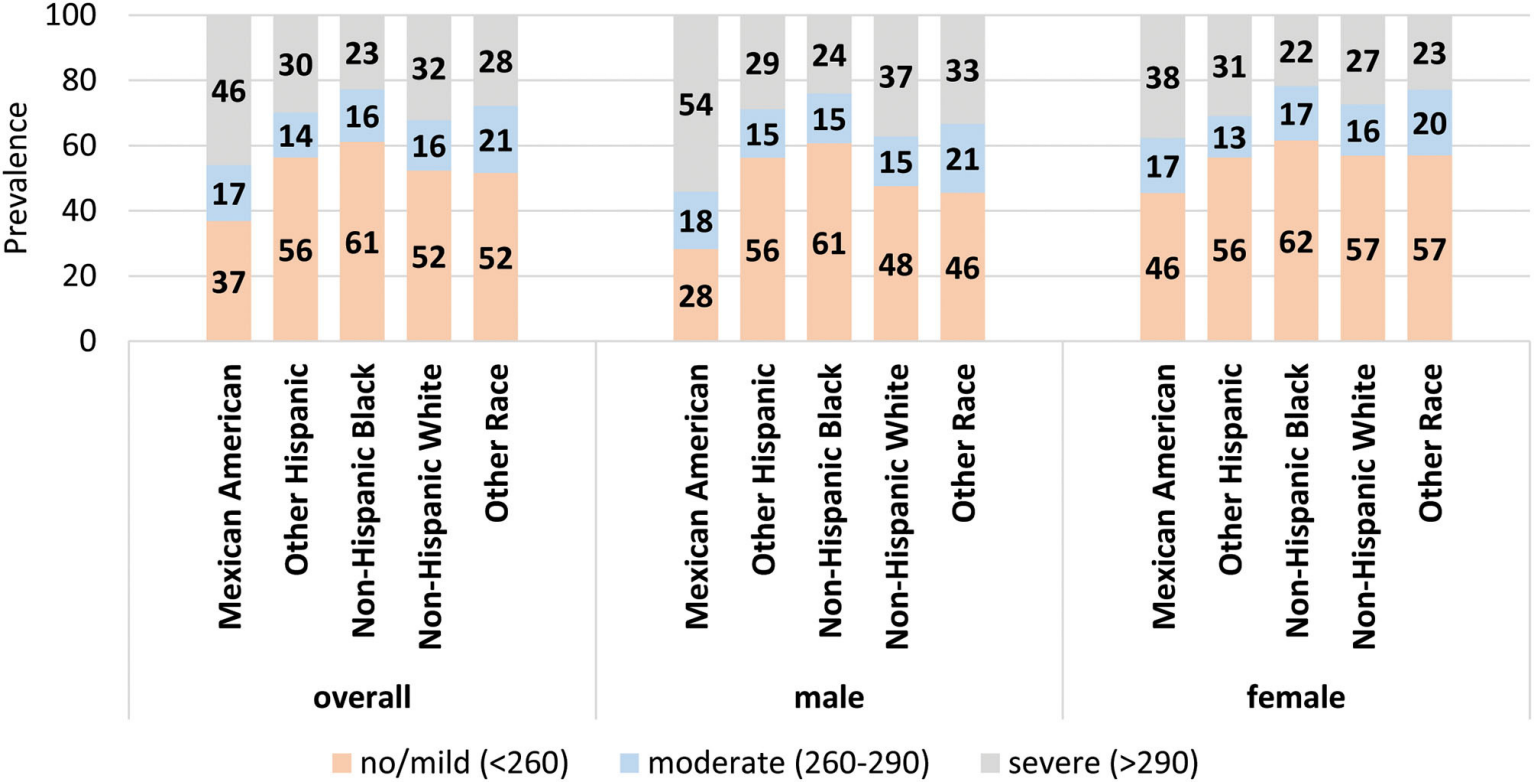
Prevalence of stages of NAFLD by race/ethnicity. The prevalence of each stage of NAFLD within each racial/ethnic group for the population overall (left) and for males and females separately (middle and right). Data labels denote the prevalence of each stage.

Overall, 52.1% had no-to-mild NAFLD, 16.1% had moderate NAFLD, and 31.8% had severe NAFLD (Table 1). The prevalence of NAFLD varied significantly by all the independent variables (*p* < 0.05) except the language spoken at home, FIR, and alcohol use (*p* > 0.05). The highest prevalence of moderate NAFLD was among subjects 65 years and older (19.3%) and that of severe NAFLD was among 50–64 years old (39.2%) (*p* < 0.05). More than one third of males (36.5%) had severe NAFLD compared to 27.4% among females (*p* < 0.05). The highest prevalence of moderate NAFLD was among the other racial/ethnic group (20.6%) and that of severe NAFLD was among Mexican Americans (46.0%) and the lowest prevalence was among Black population (22.7%) (*p* < 0.05; Figure 1). The highest prevalence of moderate NAFLD was among participants with less than high school education and of severe NAFLD was among those with some college education (35.7%) (*p* < 0.05).

Forty-four percent of subjects with high waist-to-hip ratio had severe NAFLD (*p* < 0.05). The highest prevalence of moderate NAFLD was among the overweight group (20.7%) and the highest prevalence of severe NAFLD was among the obese group (55.5%) (*p* < 0.05). Former smokers had the highest prevalence of moderate NAFLD (18.8%) and severe NAFLD (39.6%) (*p* < 0.05). About 17.6% of the physically inactive subjects had moderate NAFLD and 42.2% has severe NAFLD (*p* < 0.05). Subjects who consumed a poor-quality diet had the highest prevalence of moderate (16.8%) and severe NAFLD (33.5%). Of the subjects with high cholesterol level, 17.7% had moderate NAFLD and 37.4% had severe NAFLD (*p* < 0.05). Of those with borderline triglyceride levels, 22.6% had moderate NAFLD and 43.2% had severe NAFLD, while among those with high triglycerides, 62.5% had severe NAFLD (*p* < 0.05). The borderline HDL group had 34.8% prevalence and the low HDL group had a 56.3% prevalence of severe NAFLD. The highest prevalence of moderate NAFLD was among those with mild inflammation (19.7%) and the highest prevalence of severe NAFLD was among those with hsCRP level ≥10 mg/dL (52.4%) (*p* < 0.05). As expected, the highest prevalence of severe NAFLD was among those with elevated AST (48.1%) and ALT (53.7%) (*p* < 0.05). In the context of diabetes, moderate NAFLD was more prevalent among pre-diabetes population (18.0%) while diabetic population had a 70.6% prevalence of severe NAFLD (*p* < 0.05; Table 1).

Table 2 shows the prevalence of NAFLD stages by the independent variables among males and females. Among males, a significantly higher prevalence of moderate NAFLD was found among “other race” group (Figure 1), 65 years and older, with less than high school education, former smokers, overweight, had high waist-to-hip ratio, did not meet the guidelines for physical activity, but had cholesterol levels <200 mg/dL, 40–59 mg/dL HDL, triglyceride levels of 150–199 mg/dL, 1– <3 mg/dL CRP levels, and HbA1c levels of 5.7–6.4% (*p* < 0.05). The higher prevalence of severe NAFLD was among Mexican Americans (Figure 1), age 50–65 years of age, had some college education, obese, former smokers, physically inactive, had high waist-to-hip ratio, cholesterol levels >240 mg/dL, HDL <40 mg/dL, triglyceride >200 mg/dL, CRP >10 mg/dL, ALT >56 U/L, and HbA1c > 6.5% (*p* < 0.05).

**Table 2:** Prevalence of NAFLD stages among male and female.

	**Male**					**Female**				
	**Overall**	**No/mild (<260)**	**Moderate (260–290)**	**Severe (>290)**	***p*-value**	**Overall**	**No/mild (<260)**	**Moderate (260–290)**	**Severe (>290)**	***p*-value**
**Overall**	1,596 (48.2%)	745 (47.5%)	254 (16.0%)	597 (36.5%)		1,696 (51.8%)	923 (56.4%)	285 (16.2%)	488 (27.4%)	
**Race/ethnicity**					<0.0001					0.059
Mexican American	212 (8.8)	62 (28.3)	39 (17.6)	111 (54.1)		229 (8.1)	95 (45.5)	36 (16.8)	98 (37.7)	
Other Hispanic	140 (6.2)	66 (56.3)	23 (14.8)	51 (28.8)		146 (5.9)	81 (56.4)	21 (12.7)	44 (30.9)	
Non-Hispanic White	588 (65.7)	259 (47.6)	90 (15.3)	239 (37.1)		632 (65.6)	346 (56.9)	104 (15.7)	182 (27.4)	
Non-Hispanic Black	348 (9.5)	208 (60.7)	50 (15.3)	90 (24.0)		370 (10.3)	216 (61.6)	65 (16.7)	89 (21.6)	
Other race	308 (9.8)	150 (45.6)	52 (21.1)	106 (33.3)		319 (10.1)	185 (57.0)	59 (20.2)	75 (22.9)	
**Age (years)**					<0.0001					<0.0001
18–19	80 (3.3)	59 (81.1)	5 (5.8)	16 (13.1)		85 (3.2)	71 (86.3)	5 (5.1)	9 (8.5)	
20–34	356 (29.7)	206 (60.3)	47 (10.9)	103 (28.7)		386 (25.1)	273 (71.8)	50 (12.1)	63 (16.1)	
35–49	334 (23.7)	146 (47.3)	52 (15.9)	136 (36.8)		394 (23.6)	206 (54.6)	72 (18.8)	116 (26.5)	
50–64	447 (26.8)	172 (34.7)	85 (19.9)	190 (45.4)		475 (28.3)	205 (48.7)	88 (17.5)	182 (33.8)	
65+	379 (16.5)	162 (38.5)	65 (21.1)	152 (40.4)		356 (19.8)	168 (45.4)	70 (17.9)	118 (36.8)	
**Education**					0.068					0.039
Less than high school	322 (11.0)	153 (46.3)	54 (21.7)	115 (31.9)		254 (8.8)	133 (55.3)	41 (17.3)	80 (27.5)	
High school	406 (29.6)	200 (48.6)	61 (12.7)	145 (38.7)		415 (26.8)	221 (52.6)	59 (15.2)	135 (32.2)	
Some college	484 (29.0)	215 (45.7)	70 (12.8)	199 (41.5)		613 (32.5)	321 (52.0)	110 (17.0)	182 (30.9)	
At least college degree	384 (30.3)	177 (48.5)	69 (20.4)	138 (31.2)		414 (31.9)	248 (64.6)	75 (15.7)	91 (19.7)	
**Language spoken at home**					0.522					0.182
English	1,141 (82.2)	556 (48.2)	176 (16.4)	409 (35.4)		1,217 (83.6)	688 (57.8)	200 (15.7)	329 (26.5)	
Spanish	106 (3.5)	34 (33.3)	20 (18.0)	52 (48.6)		113 (3.5)	49 (44.7)	20 (16.8)	44 (38.5)	
Both	173 (7.7)	70 (45.2)	26 (12.2)	77 (42.6)		184 (7.1)	85 (49.8)	27 (16.2)	72 (34.0)	
Other	176 (6.6)	85 (48.4)	32 (15.4)	59 (36.2)		182 (5.9)	101 (52.7)	38 (21.6)	43 (25.7)	
**Federal income ratio (FIR)**					0.108					0.648
<1	281 (10.6)	157 (58.8)	29 (10.2)	95 (31.0)		335 (14.1)	188 (56.5)	51 (15.2)	96 (28.3)	
1–2	430 (18.4)	190 (47.2)	70 (16.1)	170 (36.7)		460 (20.7)	245 (55.5)	85 (19.5)	130 (25.0)	
>2	885 (71.1)	398 (45.9)	155 (16.9)	332 (37.2)		901 (65.2)	490 (56.7)	149 (15.3)	262 (28.0)	
**Waist-hip ratio**					<0.0001					<0.0001
Healthy	990 (64.6)	593 (62.2)	160 (14.7)	237 (23.1)		452 (29.4)	373 (86.7)	43 (6.8)	36 (6.5)	
Risk for women (≥0.85%)/risk for men (≥1.0%)	606 (35.4)	152 (20.6)	94 (18.4)	360 (61.0)		1,244 (70.6)	550 (43.8)	242 (20.0)	452 (36.1)	
BMI					<0.0001					<0.0001
Normal or healthy (<25)	416 (23.5)	350 (87.8)	35 (6.2)	31 (6.0)		518 (32.2)	443 (89.4)	48 (7.4)	27 (3.2)	
Overweight (25– <30)	586 (35.2)	280 (50.8)	130 (23.8)	176 (25.4)		465 (27.0)	264 (57.9)	78 (17.0)	123 (25.1)	
Obese (≥30)	594 (41.3)	115 (21.6)	89 (15.0)	390 (63.3)		713 (40.8)	216 (29.5)	159 (22.5)	338 (48.1)	
**Smoking status**					0.001					0.001
Current	317 (17.1)	203 (64.9)	33 (11.7)	81 (23.4)		233 (14.2)	130 (54.8)	37 (18.6)	66 (26.5)	
Former	466 (31.1)	168 (38.4)	89 (19.3)	209 (42.3)		292 (18.0)	134 (46.7)	48 (18.0)	110 (35.4)	
Non-smoker	813 (51.7)	374 (47.2)	132 (15.5)	307 (37.3)		1,171 (67.8)	659 (59.4)	200 (15.1)	312 (25.5)	
**Physical activity**					0.024					<0.0001
Inactive	294 (14.3)	113 (36.7)	45 (15.3)	136 (48.0)		439 (22.5)	205 (48.2)	88 (19.0)	146 (32.9)	
Does not meet guideline	205 (12.6)	88 (38.1)	28 (17.2)	89 (44.7)		310 (17.7)	153 (46.6)	49 (12.8)	108 (40.6)	
Meets guidelines	1,097 (73.1)	544 (51.2)	181 (16.0)	372 (32.8)		947 (59.8)	565 (62.5)	148 (16.1)	234 (21.4)	
**Healthy eating index**					0.537					<0.0001
Poor diet	1,154 (72.9)	542 (46.9)	179 (15.5)	433 (37.6)		1,150 (70.0)	615 (52.5)	200 (18.0)	335 (29.4)	
Needs improvement	404 (25.0)	185 (47.7)	68 (18.3)	151 (34.0)		483 (26.8)	275 (64.7)	72 (11.7)	136 (23.6)	
Good diet	38 (2.0)	18 (64.7)	7 (8.6)	13 (26.7)		63 (3.1)	33 (73.2)	13 (12.2)	17 (14.5)	
**Serum cholesterol**					0.006					0.125
Good (<200 mg/dL)	1,075 (65.9)	527 (50.8)	167 (16.5)	381 (32.8)		1,062 (59.7)	616 (59.7)	163 (14.9)	283 (25.4)	
Elevated (200–239 mg/dL)	374 (24.6)	163 (43.8)	64 (16.1)	147 (40.1)		451 (29.4)	221 (50.7)	83 (16.7)	147 (32.6)	
High (≥240 mg/dL)	147 (9.5)	55 (34.1)	23 (12.7)	69 (53.2)		183 (10.9)	86 (53.7)	39 (21.7)	58 (24.6)	
**High-density lipoproteins**					<0.0001					<0.0001
Low (<40 mg/dL)	410 (23.9)	118 (30.5)	69 (15.4)	223 (54.1)		146 (7.6)	39 (22.2)	22 (15.1)	85 (62.7)	
Borderline risk (40–59 mg/dL)	920 (60.7)	444 (47.9)	147 (16.9)	329 (35.1)		865 (48.8)	409 (48.7)	154 (16.9)	302 (34.4)	
Healthy (≥60 mg/dL)	266 (15.5)	183 (71.9)	38 (13.6)	45 (14.6)		685 (43.6)	475 (71.0)	109 (15.5)	101 (13.5)	
**Serum triglycerides**					<0.0001					<0.0001
Normal (<150 mg/dL)	990 (61.0)	575 (60.0)	143 (15.0)	272 (24.9)		1,246 (73.5)	795 (66.3)	196 (14.8)	255 (18.9)	
Borderline (150–199 mg/dL)	255 (16.3)	89 (33.5)	56 (21.3)	110 (45.2)		250 (15.4)	76 (34.8)	57 (24.0)	117 (41.3)	
High (≥200 mg/dL)	351 (22.6)	81 (23.7)	55 (14.9)	215 (61.4)		200 (11.1)	52 (21.2)	32 (14.1)	116 (64.7)	
**High-sensitivity CRP**					<0.0001					<0.0001
Normal (0.1– <1 mg/dL)	522 (33.1)	331 (65.8)	72 (13.1)	119 (21.1)		482 (29.4)	374 (78.4)	65 (11.8)	43 (9.9)	
Mild inflammation (1– <3 mg/dL)	635 (40.8)	265 (42.2)	115 (19.7)	255 (38.0)		536 (31.8)	294 (57.4)	102 (19.6)	140 (23.0)	
Significant inflammation (3– <10 mg/dL)	370 (21.9)	125 (33.1)	52 (14.4)	193 (52.5)		514 (29.6)	203 (40.9)	92 (16.4)	219 (42.8)	
High significant inflammation (≥10 mg/dL)	69 (4.2)	24 (29.1)	15 (11.4)	30 (59.4)		164 (9.1)	52 (33.1)	26 (17.5)	86 (49.4)	
**Aspartate aminotransferase (AST)**					0.452					0.012
Normal (≤ 40 U/L)	1,518 (94.3)	714 (47.9)	247 (16.3)	557 (35.8)		1,647 (97.7)	903 (56.9)	277 (16.2)	467 (26.9)	
Elevated (>40 U/L)	78 (5.7)	31 (40.4)	7 (11.6)	40 (48.0)		49 (2.3)	20 (37.0)	8 (14.8)	21 (48.2)	
**Alanine aminotransferase (ALT)**					0.062					0.022
Normal (≤ 56 U/L)	1,513 (94.3)	722 (48.5)	244 (16.2)	547 (35.4)		1,666 (98.4)	912 (56.7)	282 (16.3)	472 (27.0)	
Elevated (>56 U/L)	83 (5.7)	23 (31.1)	10 (13.9)	50 (55.0)		30 (1.6)	11 (40.1)	3 (10.2)	16 (49.8)	
**Hemoglobin A1c (HbA1c%)**					<0.0001					<0.0001
Healthy (<5.7%)	876 (66.5)	499 (57.7)	126 (15.6)	251 (26.7)		1,026 (67.9)	695 (66.9)	161 (16.0)	170 (17.1)	
Pre-diabetes (5.7–6.4%)	489 (23.7)	187 (31.5)	97 (18.4)	205 (50.1)		464 (23.8)	191 (41.0)	88 (17.7)	185 (41.3)	
Diabetes (≥6.5%)	231 (9.9)	59 (17.2)	31 (13.3)	141 (69.5)		206 (8.3)	37 (15.2)	36 (12.9)	133 (71.9)	

Among females, significantly higher prevalence of moderate NAFLD was found among 65 years and older, with less than high school education, current smokers, obese, had high waist-to-hip ratio, physically inactive, ate poor-quality diet, had HDL 40–59 mg/dL, triglyceride levels of 150–199 mg/dL, CRP levels of 1– <3 mg/dL, AST <40 U/L, ALT <56 U/L, and HbA1c levels of 5.7–6.4% (*p* < 0.05). The higher prevalence of severe NAFLD was among 65 years and older, had high school education, obese, former smokers, did not meet physical activity guideline, eat poor quality diet, had high waist -to-hip ratio, HDL <40 mg/dL, triglyceride >200 mg/dL, CRP >10 mg/dL, AST >40 U/L, ALT >56 U/L, and HbA1c >6.5% (*p* < 0.05).

**Figure 2:**
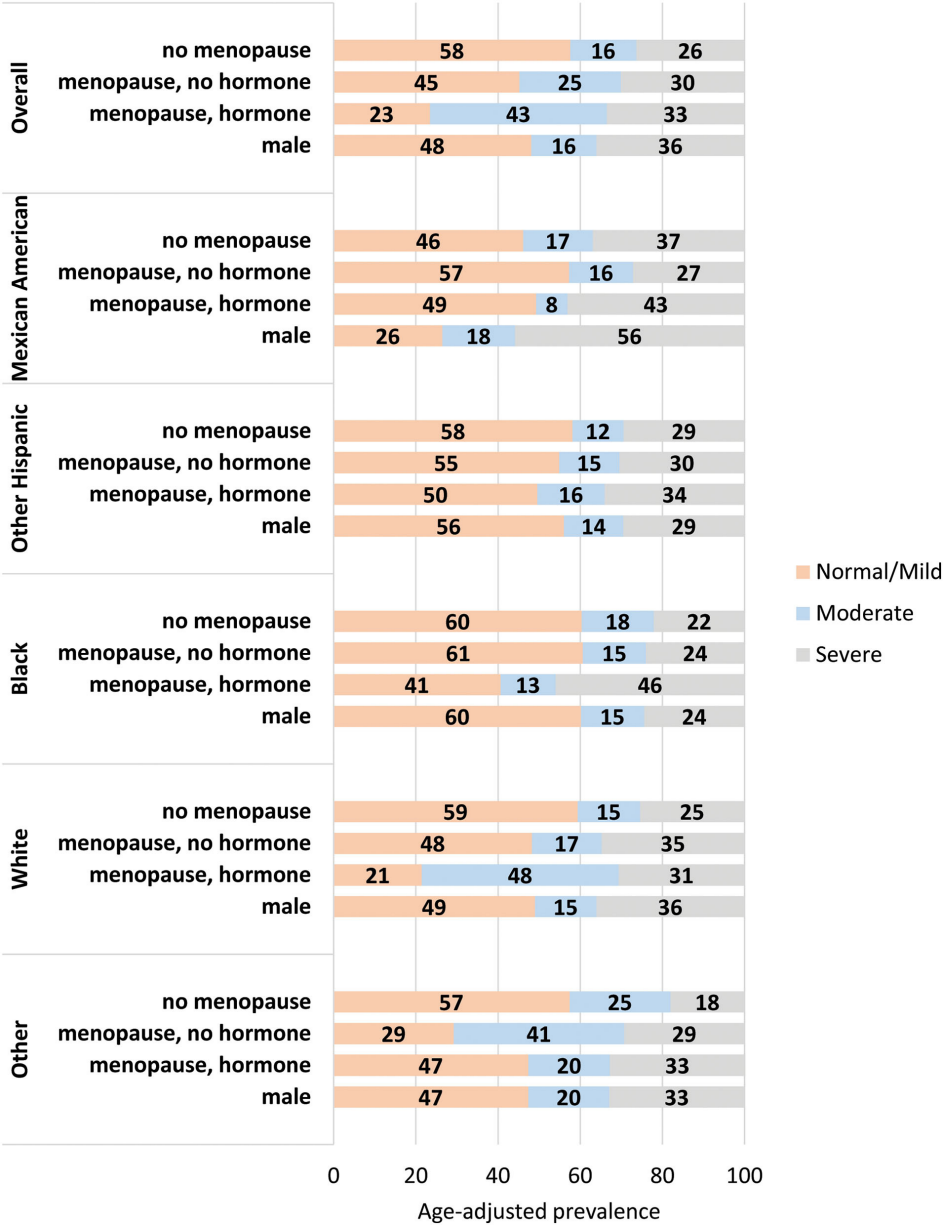
Age-adjusted prevalence of stages of NAFLD by gender and menopausal status. The age-adjusted prevalence of each stage of NAFLD within each gender/menopause group for the population overall (top) and stratified by racial/ethnic group. Data labels denote the prevalence of each stage.

Table 3 and Figure 2 show the age-adjusted prevalence of NAFLD stage by race/ethnicity, gender and menopausal status with and without female hormone therapy. Of the 1,696 females, 510 were menopausal (out of these, 110 females used hormone therapy). Overall, the age adjusted prevalence of moderate NAFLD was highest among menopausal females who used hormone therapy (43.1%) followed by menopausal females with no hormone therapy (24.7%), then non-menopausal females (16.1%) and males (15.9%) (*p* < 0.05). Overall, the age adjusted prevalence of severe NAFLD was highest among males (36%), followed by menopausal females who used hormone therapy (33.5%), followed by menopausal females with no hormone therapy (30.1%), then non-menopausal females (26.3%) (*p* < 0.05). Among Mexican Americans, significantly higher prevalence of severe NAFLD was found in males (55.8%), followed by menopausal females with hormone therapy (43.1%). Among other Hispanics and Blacks, severe NAFLD was higher among menopausal females who used hormone therapy (34.0 and 45.9%, respectively). Among Whites and other race/ethnicity groups, higher prevalence of severe NAFLD was found among males (36.0 and 32.9%, respectively) relative to females (Table 3).

**Table 3:** Age-adjusted prevalence of NAFLD stage by race/ethnicity and gender.

**Variable**	**Male (*n* = 1,596)**	**Female no menopause (*N* = 1,186)**	**Female menopause no hormone (*N* = 400)**	**Female menopause plus hormone (*N* = 110)**	***p*-value**
**Overall**					<0.001
Normal/mild	745 (48.12)	689 (57.60)	179 (45.20)	55 (23.44)	
Moderate	254 (15.86)	193 (16.07)	70 (24.72)	22 (43.08)	
Severe	597 (36.01)	304 (26.32)	151 (30.08)	33 (33.47)	
**Mexican American**					<0.001
Normal/mild	62 (26.45)	80 (46.10)	12 (57.30)	3 (49.31)	
Moderate	39 (17.70)	26 (16.97)	9 (15.57)	1 (7.60)	
Severe	111 (55.83)	67 (36.92)	25 (27.14)	6 (43.09)	
**Other Hispanics**					0.9945
Normal/mild	66 (56.04)	53 (58.11)	21 (54.90)	7 (49.57)	
Moderate	23 (14.49)	11 (12.43)	8 (14.66)	2 (16.40)	
Severe	51 (29.45)	27 (29.45)	12 (30.45)	5 (34.03)	
**Blacks**					0.4346
Normal/mild	208 (60.19)	164 (60.38)	41 (60.67)	11 (40.59)	
Moderate	50 (15.37)	47 (17.55)	14 (15.32)	4 (13.48)	
Severe	90 (24.43)	53 (22.07)	34 (24.03)	2 (45.93)	
**Whites**					<0.001
Normal/mild	259 (49.02)	253 (59.36)	69 (48.28)	24 (21.46)	
Moderate	90 (14.95)	68 (15.19)	24 (16.95)	12 (47.91)	
Severe	239 (36.01)	114 (25.45)	51 (34.77)	17 (30.62)	
**Others**					0.0083
Normal/mild	150 (47.37)	139 (57.40)	36 (29.23)	10 (47.37)	
Moderate	52 (19.73)	41 (24.59)	15 (41.49)	3 (19.89)	
Severe	106 (32.89)	43 (18.00)	29 (29.28)	3 (32.89)	

### Factors Associated With NAFLD Stages

In the multinomial adjusted model (Table 4), race/ethnicity was significantly associated with NAFLD stage. Mexican Americans were two times more likely to have moderate NAFLD [adjusted odds ratio (AOR) = 1.9, 95% confidence interval (CI) = 1.1–3.4, *p* < 0.05] and were more than twice as likely to have severe NAFLD (AOR = 2.4, 95% CI = 1.4–4.2, *p* < 0.05) relative to the White population. In addition, the Black population was significantly less likely to have severe NAFLD relative to White population (AOR = 0.5, 95% CI = 0.4–0.7, *p* < 0.05).

**Table 4:** Adjusted odds ratio (OR) and 95% confidence interval (CI) for the relationship between NAFLD and race/ethnicity (reference = no/mild NAFLD).

**Reference = no/mild NAFLD**	**Moderate (260–290)**		**Severe (>290)**	
	**AOR [95% CI]**	***p*-value**	**AOR [95% CI]**	***p*-value**
**Race/Ethnicity**				
Mexican-American vs. non-Hispanic White	**1.9 [1.1–3.4]**	**0.0178**	**2.4 [1.4–4.2]**	**0.0015**
Other Hispanic vs. non-Hispanic White	0.9 [0.5–1.4]	0.5159	0.7 [0.4–1.2]	0.1807
Non-Hispanic Black vs. non-Hispanic White	0.9 [0.6–1.3]	0.5245	**0.5 [0.4–0.7]**	**<0.0001**
Other race vs. non-Hispanic White	1.7 [0.9–3.1]	0.1010	1.0 [0.6–1.8]	0.9419
**Age (years)**				
18–19 vs. 20–34	0.6 [0.3–1.3]	0.1904	0.6 [0.3–1.2]	0.1671
35–49 vs. 20–34	1.4 [1.0–2.1]	0.0723	1.2 [0.8–1.7]	0.3900
50–64 vs. 20–34	**2.3 [1.3–4.3]**	**0.0059**	**2.1 [1.4–3.1]**	**0.0007**
65+ vs. 20–34	**2.0 [1.1–3.7]**	**0.0346**	1.6 [0.9–2.7]	0.0932
**Education**				
Less than high school vs. high school	**1.4 [1.0–2.0]**	**0.0373**	0.7 [0.4–1.2]	0.2138
Some college vs. high school	1.2 [0.7–2.0]	0.5723	1.1 [0.7–1.9]	0.6944
At least college degree vs. high school	1.4 [0.8–2.7]	0.2785	0.9 [0.5–1.6]	0.7864
**Language spoken at home**				
Spanish vs. English	0.8 [0.5–1.4]	0.4419	0.8 [0.5–1.4]	0.4560
Both vs. English	0.8 [0.5–1.4]	0.4874	1.1 [0.6–2.0]	0.8350
Other vs. English	1.2 [0.6–2.5]	0.5989	1.5 [0.8–3.0]	0.2312
**Federal income ratio (FIR)**				
<1 vs. >2	0.8 [0.6–1.1]	0.2188	0.8 [0.6–1.2]	0.2640
1–2 vs. >2	1.1 [0.7–1.8]	0.6418	0.9 [0.6–1.2]	0.3799
**Waist-hip ratio**				
Risk for women (≥0.85)/risk for men (≥1.0) vs. healthy	**1.7 [1.1–2.8]**	**0.0249**	**1.8 [1.2–2.7]**	**0.0041**
**BMI**				
Overweight (25– <30) vs. normal (<25)	**3.9 [2.4–6.6]**	**<0.0001**	**7.1 [3.9–13.1]**	**<0.0001**
Obese (≥30) vs. normal (<25)	**6.5 [3.7–11.6]**	**<0.0001**	**24.8 [11.9–51.9]**	**<0.0001**
**Smoking status**				
Current vs. never	0.9 [0.6–1.4]	0.5794	0.7 [0.5–1.1]	0.1166
Former vs. never	1.2 [0.9–1.6]	0.3316	1.2 [0.9–1.7]	0.2279
**Alcohol use**				
Current vs. never	1.4 [0.9–2.3]	0.1269	0.7 [0.4–1.2]	0.1888
Former vs. never	1.4 [0.9–2.3]	0.1717	0.6 [0.3–1.1]	0.1136
**Physical activity**				
Inactive vs. meets guidelines	1.0 [0.8–1.4]	0.8087	1.2 [0.9–1.8]	0.2674
Does not meet guidelines vs. meets guidelines	0.9 [0.5–1.6]	0.6670	1.4 [0.9–2.2]	0.1557
**Healthy eating index**				
Poor diet vs. good diet	1.6 [0.7–3.9]	0.2610	0.9 [0.2–3.9]	0.9219
Needs improvement vs. good diet	1.2 [0.5–3.3]	0.6525	0.9 [0.2–3.4]	0.8771
**Serum cholesterol**				
Elevated (200–239 mg/dL) vs. good (<200 mg/dL)	0.8 [0.5–1.2]	0.2176	0.9 [0.7–1.3]	0.7105
High (≥240 mg/dL) vs. good (<200 mg/dL)	0.8 [0.6–1.1]	0.1756	0.8 [0.6–1.1]	0.1753
**High-density lipoproteins**				
Low (<40 mg/dL) vs. healthy (≥60 mg/dL)	1.2 [0.6–2.7]	0.5843	**2.7 [1.6–4.7]**	**0.0003**
Borderline risk (40–59 mg/dL) vs. healthy (≥60 mg/dL)	1.2 [0.8–1.7]	0.4582	**1.8 [1.2–2.6]**	**0.0032**
**Serum triglycerides**				
Borderline (150–199 mg/dL) vs. normal (<150 mg/dL)	**1.8 [1.3–2.4]**	**0.0009**	**1.7 [1.0–2.8]**	**0.0375**
High (≥200 mg/dL) vs. normal (<150 mg/dL)	**1.7 [1.3–2.4]**	**0.0007**	**2.9 [1.9–4.4]**	**<0.0001**
**High-sensitivity CRP**				
Mild inflammation (1–3 mg/dL) vs. normal (<1 mg/dL)	1.4 [0.9–2.2]	0.1638	1.2 [0.7–2.0]	0.4566
Significant inflammation (3–10 mg/dL) vs. normal (<1 mg/dL)	1.1 [0.7–1.7]	0.7856	1.4 [0.8–2.5]	0.2684
High significant inflammation (≥10 mg/dL)	1.3 [0.8–2.0]	0.2317	1.8 [0.8–4.0]	0.1597
**Aspartate aminotransferase (AST)**				
Elevated (>40 U/L) vs. normal (≤ 40 U/L)	1.4 [0.3–6.3]	0.6815	1.9 [0.7–5.0]	0.2018
**Alanine aminotransferase (ALT)**				
Elevated (>56 U/L) vs. normal (≤ 56 U/L)	1.3 [0.3–4.6]	0.7310	1.9 [0.6–6.2]	0.2611
**Hemoglobin A1c (HbA1c)**				
Pre-diabetes vs. healthy (<5.7%)	1.3 [0.9–1.9]	0.2221	**2.4 [1.7–3.6]**	**<0.0001**
Diabetes (≥6.5%) vs. healthy (<5.7%)	1.6 [0.9–2.8]	0.1469	**5.0 [2.6–9.3]**	**<0.0001**

*Bold = statistically significant at p < 0.05*.

Relative to the age group of 20–34 years of age, the 50–64 years and 65+ years old groups were about two times more likely to have moderate NAFLD; the 50–64 years old group was also two times more likely to have severe NAFLD (*p* < 0.05). Participants in the high-risk group of the waist-to-hip ratio were about two times more likely to have moderate NAFLD and to have severe NAFLD (*p* < 0.05) relative to those in the normal waist-to-hip ratio group. Relative to the group with normal BMI, overweight participants were 3.9 times more likely to have moderate NAFLD and about seven times more likely to have severe NAFLD (<0.05). This relationship was stronger among the obese population for both moderate NAFLD (six times higher odds) and severe NAFLD (25 times higher odds) (*p* < 0.05).

Participants with borderline and low HDL levels had higher odds of developing severe NAFLD than those with a normal level of HDL (*p* < 0.05). Participants with borderline and high levels of triglycerides had higher odds than those with normal levels to have moderate and severe NAFLD (*p* < 0.05). Subjects with high HbA1c in both groups of pre-diabetes and diabetes had higher odds of severe NAFLD relative to the normal group (*p* < 0.05). Patients with pre-diabetes had twice the odds of developing severe NAFLD (*p* < 0.05) and patients with diabetes had 5 times higher odds of displaying severe NAFLD relative to the normal group (Table 4).

**Figure 3:**
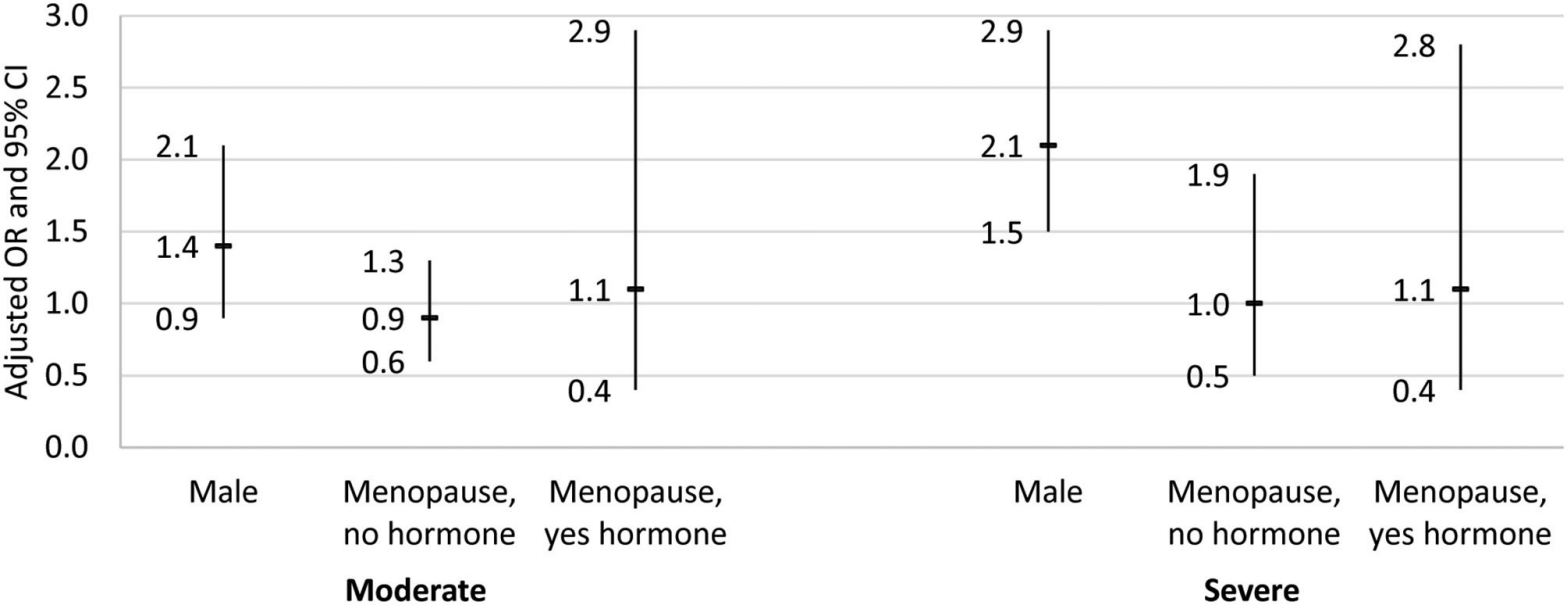
Adjusted odds ratio and 95% confidence interval for the association between gender/menopause group and NAFLD stage. Results from the multinomial logistic regression for the association between gender/menopause group and NAFLD stage. Dash indicates odds ratio, and bar denotes the 95% confidence interval. Reference group for the dependent variable was no/mild NAFLD, and the reference group for gender/menopause was female, no menopause. Regression adjusted for demographic variables (age, race/ethnicity, education, language spoken, and poverty), physical activity status, smoking status, diet quality (healthy eating index), body composition (waist-to-hip ratio and body mass index), and laboratory values [cholesterol, HDL, triglyceride, glucose, hemoglobin A1c (HbA1c), highly-sensitive C-reactive protein (hsCRP), AST, and ALT].

Table 5 and Figure 3 shows that after adjustment for the other independent variables, males had two times higher odds of severe NAFLD relative to non-menopausal females (AOR = 2.1, 95% CI = 1.5–2.9, *p* < 0.05). There was no difference in the likelihood of having moderate or severe NAFLD in the menopausal females with or without hormone therapy relative to non-menopausal females (*p* > 0.05).

**Table 5:** Adjusted odds ratio (AOR) and 95% confidence interval (CI) of the association between NAFLD stage and gender.

**Reference = normal/mild NAFLD**	**NAFLD**	
	**Moderate**	**Severe**
	**AOR [95% CI]**	**AOR [95% CI]**
No menopause	Ref	Ref
Menopause, no hormone	0.9 [0.6–1.3]	1.0 [0.5–1.9]
Menopause, yes hormone	1.1 [0.4–2.9]	1.1 [0.4–2.8]
Male	1.4 [0.9–2.1]	**2.1 [1.5–2.9]**

*Adjusted for demographic variables (age, race/ethnicity, education, language spoken, and poverty), physical activity status, smoking status, diet quality (healthy eating index), body composition (waist-to-hip ratio and body mass index), and laboratory values [cholesterol, HDL, triglyceride, glucose, hemoglobin A1c (HbA1c), highly-sensitive C-reactive protein (hsCRP), AST, and ALT]*.

*Bold = statistically significant at p < 0.05*.

### Gender Is an Effect Modifier of the Association Between Race/Ethnicity and NAFLD Stages

In the stratified analysis (Table 6), after adjusting for the other independent variables, Mexican Americans had more than four times higher odds of moderate and severe NAFLD relative to Whites only among males (*p* < 0.05) and similar odds as Whites among females (*p* > 0.05). Whereas, non-Hispanic Blacks had lower odds of severe NAFLD relative to Whites among both males and females, after adjusting for other independent variables (*p* < 0.05).

**Table 6:** Adjusted odds ratio (AOR) and 95% confidence interval (CI) for the relationship between NAFLD and race/ethnicity by gender (reference = no/mild NAFLD).

**Reference = no/mild NAFLD**	**Male**				**Female**			
	**Moderate (260–290)**		**Severe (>290)**		**Moderate (260–290)**		**Severe (>290)**	
	**AOR [95% CI]**	***p*-value**	**AOR [95% CI]**	***p*-value**	**AOR [95% CI]**	***p*-value**	**AOR [95% CI]**	***p*-value**
**Race/Ethnicity**								
Mexican Americans vs. non-Hispanic White	**4.5 [1.7–11.7]**	**0.0020**	**5.0 [2.1–12.0]**	**0.0003**	0.9 [0.4–2.3]	0.8628	1.8 [0.6–5.8]	0.3296
Other Hispanic vs. non-Hispanic White	1.2 [0.5–2.6]	0.7152	0.7 [0.3–2.2]	0.5927	0.5 [0.2–1.6]	0.2700	0.9 [0.3–2.9]	0.9131
Non-Hispanic Black vs. non-Hispanic White	1.0 [0.5–1.9]	0.9972	**0.5 [0.3–0.9]**	**0.0212**	0.7 [0.5–1.1]	0.1076	**0.5 [0.3–0.9]**	**0.0101**
Other race vs. non-Hispanic White	2.3 [0.9–5.9]	0.0788	1.3 [0.7–2.6]	0.3878	1.2 [0.6–2.5]	0.6419	0.8 [0.4–1.9]	0.6667
**Age (years)**								
18–19 vs. 20–34	0.6 [0.2–2.0]	0.3815	0.6 [0.3–1.3]	0.2346	0.6 [0.1–2.7]	0.5121	0.6 [0.2–2.2]	0.4404
35–49 vs. 20–34	1.3 [0.6–2.9]	0.5466	1.1 [0.6–2.0]	0.7388	1.6 [0.8–3.2]	0.2128	1.6 [0.7–3.5]	0.2865
50–64 vs. 20–34	**2.8 [1.5–5.6]**	**0.0023**	**2.3 [1.2–4.3]**	**0.0111**	1.9 [0.9–3.8]	0.0747	**2.4 [1.4–4.0]**	**0.0014**
65+ vs. 20–34	**2.4 [1.1–5.1]**	**0.0202**	1.4 [0.7–2.8]	0.3110	1.7 [0.7–4.1]	0.2375	**2.2 [1.1–4.7]**	**0.0324**
**Education**								
Less than high school vs. high school	**2.2 [1.1–4.2]**	**0.0196**	0.8 [0.5–1.4]	0.4710	1.0 [0.6–1.7]	0.8996	0.6 [0.2–1.5]	0.2680
Some college vs. high school	0.9 [0.5–1.5]	0.6436	1.0 [0.5–2.0]	0.9845	1.5 [0.9–2.8]	0.1479	1.4 [0.8–2.4]	0.2869
At least college degree vs. high school	1.3 [0.6–2.7]	0.5380	0.8 [0.5–1.3]	0.3531	1.4 [0.6–3.0]	0.4505	1.0 [0.4–2.6]	0.9502
**Language spoken at home**								
Spanish vs. English	0.4 [0.1–1.1]	0.0634	0.4 [0.1–1.4]	0.1506	2.1 [0.6–7.6]	0.2758	1.1 [0.2–5.6]	0.8652
Both vs. English	0.3 [0.1–1.0]	0.0546	0.6 [0.2–2.2]	0.4489	2.1 [0.8–5.6]	0.1429	1.3 [0.4–4.2]	0.6568
Other vs. English	0.8 [0.4–1.6]	0.4946	1.7 [0.6–4.4]	0.3139	1.8 [0.7–4.6]	0.2155	1.7 [0.8–3.7]	0.1983
**Federal income ratio (FIR)**								
<1 vs. >2	0.7 [0.3–1.4]	0.2760	0.9 [0.5–1.8]	0.8686	0.9 [0.5–1.6]	0.6853	0.8 [0.5–1.4]	0.4980
1–2 vs. >2	1.2 [0.8–1.7]	0.4381	1.3 [0.7–2.4]	0.3633	1.1 [0.6–2.0]	0.8161	0.7 [0.4–1.1]	0.0815
**Waist-hip ratio**								
Risk for women (≥0.85)/risk for men (≥1.0) vs. healthy	1.6 [0.8–2.9]	0.1518	**1.9 [1.2–3.0]**	**0.0036**	**3.0 [1.3–6.8]**	**0.0073**	**2.9 [1.4–6.0]**	**0.0042**
**BMI**								
Overweight (25– <30) vs. normal (<25)	**5.3 [2.9–9.6]**	**<0.0001**	**5.9 [3.2–11.2]**	**<0.0001**	**2.5 [1.3–4.8]**	**0.0053**	**8.1 [4.3–15.3]**	**<0.0001**
Obese (≥30) vs. normal (<25)	**6.9 [3.3–14.6]**	**<0.0001**	**30.6 [14.1–66.3]**	**<0.0001**	**6.4 [3.4–12.0]**	**<0.0001**	**21.0 [7.7–57.6]**	**<0.0001**
**Smoking status**								
Current vs. never	0.5 [0.2–1.4]	0.1854	**0.5 [0.2–0.9]**	**0.0209**	1.2 [0.8–1.8]	0.4637	1.0 [0.6–1.6]	0.9229
Former vs. never	1.0 [0.6–1.8]	0.8638	1.0 [0.7–1.6]	0.8662	1.0 [0.6–1.8]	0.9425	1.0 [0.7–1.6]	0.9411
**Alcohol use**								
Current vs. never	1.2 [0.7–2.4]	0.5136	0.6 [0.3–1.3]	0.2230	1.4 [0.8–2.4]	0.2807	0.7 [0.3–1.5]	0.3233
Former vs. never	1.1 [0.4–2.9]	0.8811	**0.4 [0.2–0.9]**	**0.0278**	1.4 [0.6–3.1]	0.4336	0.7 [0.3–1.6]	0.4653
**Physical activity**								
Inactive vs. meets guidelines	1.1 [0.5–2.2]	0.7924	**1.9 [1.1–3.4]**	**0.0258**	1.0 [0.6–1.6]	0.9031	1.1 [0.6–2.1]	0.7934
Does not meet guidelines vs. meets guidelines	1.0 [0.5–2.1]	0.9900	1.3 [0.7–2.3]	0.3704	0.8 [0.4–1.5]	0.5128	1.8 [0.9–3.6]	0.0707
**Healthy eating index**								
Poor diet vs. good diet	2.6 [0.6–10.8]	0.1841	0.9 [0.1–7.7]	0.9529	1.2 [0.3–3.9]	0.8100	0.9 [0.3–3.0]	0.9028
Needs improvement vs. good diet	2.5 [0.6–9.7]	0.1947	1.2 [0.2–9.0]	0.8682	0.7 [0.2–2.1]	0.5085	0.7 [0.3–1.9]	0.4773
**Serum cholesterol**								
Elevated (200–239 mg/dL) vs. good (<200 mg/dL)	**0.6 [0.4–0.9]**	**0.0243**	0.6 [0.3–1.1]	0.1125	1.1 [0.7–1.6]	0.7665	1.2 [0.8–2.0]	0.3699
High (≥240 mg/dL) vs. good (<200 mg/dL)	0.5 [0.2–1.2]	0.1320	0.9 [0.4–1.9]	0.8289	1.1 [0.6–1.8]	0.8074	0.6 [0.3–1.1]	0.0947
**High-density lipoproteins**								
Low (<40 mg/dL) vs. healthy (≥60 mg/dL)	1.0 [0.4–2.6]	0.9782	1.8 [0.8–3.8]	0.1262	1.5 [0.3–6.8]	0.6300	**3.7 [1.4–9.5]**	**0.0067**
Borderline risk (40–59 mg/dL) vs. healthy (≥60 mg/dL)	1.2 [0.7–2.1]	0.5357	1.6 [0.8–3.3]	0.1722	0.9 [0.6–1.5]	0.7587	1.4 [0.9–2.2]	0.1465
**Serum triglycerides**								
Borderline (150–199 mg/dL) vs. normal (<150 mg/dL)	1.9 [1.0–3.5]	0.0594	1.7 [0.7–4.0]	0.2692	**1.9 [1.2–2.8]**	**0.0035**	**1.9 [1.2–3.3]**	**0.0121**
High (≥200 mg/dL) vs. normal (<150 mg/dL)	**2.3 [1.2–4.2]**	**0.0074**	**3.1 [1.7–5.6]**	**0.0003**	1.3 [0.5–3.7]	0.5616	**3.3 [1.9–5.5]**	**<0.0001**
**High-sensitivity CRP**								
Mild inflammation (1–3 mg/dL) vs. normal (<1 mg/dL)	1.8 [1.0–3.2]	0.0562	1.4 [0.8–2.6]	0.2520	1.2 [0.7–2.0]	0.5222	1.1 [0.6–2.0]	0.8330
Significant inflammation (3–10 mg/dL) vs. normal (<1 mg/dL)	1.4 [0.7–3.0]	0.3518	1.5 [0.7–3.5]	0.3001	1.0 [0.5–1.9]	0.9679	1.8 [0.8–4.2]	0.1633
High significant inflammation (≥10 mg/dL) vs. normal	1.1 [0.5–2.4]	0.7610	2.2 [0.7–6.9]	0.1900	1.2 [0.6–2.4]	0.5629	2.5 [0.9–6.7]	0.0697
**Aspartate aminotransferase (AST)**								
Elevated (>40 U/L) vs. normal (≤ 40 U/L)	1.0 [0.1–8.2]	0.9759	1.3 [0.6–2.8]	0.5321	**4.2 [1.0–17.5]**	**0.0485**	**5.4 [1.3–22.5]**	**0.0193**
**Alanine aminotransferase (ALT)**								
Elevated (>56 U/L) vs. normal (≤ 56 U/L)	2.5 [0.6–11.5]	0.2292	**3.0 [1.1–8.3]**	**0.0389**	**0.1 [0.0–0.8]**	**0.0285**	0.2 [0.0–1.1]	0.0669
**Hemoglobin A1c (HbA1c)**								
Pre-diabetes vs. healthy (<5.7%)	1.5 [0.9–2.3]	0.1244	**2.7 [1.7–4.4]**	**<0.0001**	1.2 [0.7–2.2]	0.4679	**2.2 [1.3–3.5]**	**0.0019**
Diabetes (≥6.5%) vs. healthy (<5.7%)	1.3 [0.5–3.3]	0.6118	**3.8 [1.4–10.5]**	**0.0097**	**2.0 [1.0–3.9]**	**0.0428**	**7.0 [3.8–13.0]**	**<0.0001**

*Bold = statistically significant at p < 0.05*.

### Factors Associated With Moderate NAFLD Stages Among Males and Females

In the stratified multinomial adjusted model (Table 6), NAFLD was significantly associated with old age, a high waist-to-hip ratio, a high BMI, borderline/high triglyceride level, and pre-diabetes and diabetes diagnosis by HbA1c in both genders (*p* < 0.05). While age was not associated with moderate NAFLD in females (*p* > 0.05), males aged 50 years and older had over two-fold chances of developing moderate NAFLD relative to those of 20–34 years of age (*p* < 0.05). Waist-to-hip ratio was not associated with moderate NAFLD among males (*p* > 0.05), but females with high waist-to-hip ratio were about three times more likely to have moderate NAFLD relative to females with a normal waist-to-hip ratio (*p* < 0.05). Those who were overweight or obese were more likely than the normal group to have moderate NAFLD among both males and females (*p* < 0.05). While borderline triglyceride level was associated with moderate NAFLD among only females, high triglyceride level was associated with moderate NAFLD among males only (*p* < 0.05). High AST and ALT levels as well as the level of HbA1c were associated with moderate NAFLD only among females (*p* < 0.05) and not among males (*p* > 0.05; Table 6).

Table 7 shows that among males, Mexican Americans had more than 4 times higher odds of moderate NAFLD relative to Whites (AOR = 4.5 [95% CI = 1.7–11.7], *p* < 0.05). There was no association between race/ethnicity and moderate NAFLD among females with and without menopause (*p* > 0.05).

**Table 7:** Adjusted odds ratio (AOR) and 95% confidence interval (CI) of the association between NAFLD stage and race/ethnicity for each gender group.

**Reference: normal/mild NAFLD**	**Male**		**Female no menopause**		**Female menopause no hormone**	
**Race/ethnicity**	**Moderate**	**Severe**	**Moderate**	**Severe**	**Moderate**	**Severe**
Non-Hispanic White	Ref	Ref	Ref	Ref	Ref	Ref
Mexican American	**4.5 [1.7–11.7]**	**5.0 [2.1–12.0]**	0.8 [0.2–3.0]	2.3 [0.6–8.2]	2.7 [0.2–50.0]	2.5 [0.2–34.4]
Other Hispanic	1.2 [0.5–2.6]	0.7 [0.3–2.2]	0.6 [0.1–2.3]	1.8 [0.7–4.8]	0.2 [0.0–4.7]	0.4 [0.0–12.2]
Non-Hispanic Black	1.0 [0.5–1.9]	**0.5 [0.3–0.9]**	0.7 [0.4–1.2]	**0.5 [0.3–0.9]**	0.7 [0.2–1.9]	0.6 [0.2–1.7]
Other race	2.3 [0.9–6.0]	1.3 [0.7–2.6]	1.0 [0.5–2.3]	0.8 [0.3–2.5]	3.6 [1.0–13.2]	2.0 [0.5–7.8]

*Adjusted for demographic variables (age, race/ethnicity, education, language spoken, and poverty [FIR]), physical activity status, smoking status, diet quality (healthy eating index), body composition (waist-to-hip ratio and body mass index), and laboratory values [cholesterol, HDL, triglyceride, glucose, hemoglobin A1c (HbA1c), highly-sensitive C-reactive protein (hsCRP), AST, and ALT]*.

*Bold = statistically significant at p < 0.05*.

### Factors Associated With Severe NAFLD Stages Among Males and Females

Severe NAFLD was associated with 50–64 years of age, high waist-to-hip ratio, high BMI, high levels of triglycerides, and pre-diabetes and diabetes status among both males and females (*p* < 0.05; Table 6). While females aged 65 years and older had more than two times higher odds of severe NAFLD (*p* < 0.05), this relation was not statistically significant among males (*p* > 0.05). For males, current smokers had lower odds of having severe NAFLD than those who never smoked (*p* > 0.05); this relationship was not observed in females (*p* > 0.05). While physically inactive males had about twice the odds to develop severe NAFLD relative to physically active males (*p* < 0.05), physical inactivity did not play a significant role in females (*p* > 0.05). While females with low HDL levels had about four times higher odds of severe NAFLD relative to females with normal HDL (*p* < 0.05), this association was not statistically significant among males (*p* > 0.05). In females, both borderline and high levels of triglycerides increased the odds of severe NAFLD, but in males only high levels increased the odds. In addition, females with high level of AST level had about five times higher odds of severe NAFLD relative to females with normal AST level (*p* < 0.05), but this association was not statistically significant among males (*p* > 0.05). In males, on the other hand, a high level of ALT was associated with a three times higher odds of severe NAFLD relative to males with a normal ALT level (*p* < 0.05); this association was not significant in females (*p* > 0.05; Table 6).

Table 7 shows that among males, Mexican Americans had five times higher odds of severe NAFLD relative to Whites (AOR = 5.0 [95% CI = 2.1–12.0], *p* < 0.05). Blacks had lower odds of severe NAFLD relative to Whites among males and non-menopausal females (AOR = 0.5 [95% CI = 0.3–0.9], *p* < 0.05 and AOR = 0.5 [95% CI = 0.3–0.9], *p* < 0.05).

## Discussion

The current study analyzed data from the NHANES 2017–2018 database and examined the association of gender with racial/ethnic differences with respect to NAFLD stages in the US population (normal/mild, moderate, and severe), as estimated by transient elastography. More than half of participants had no/mild NAFLD, with about one third having severe NAFLD. The findings from our study are consistent with other studies showing that ~1 in 3 subjects had NAFLD (12) with CAP> 290 dB/m. Importantly, Mexican Americans had the highest prevalence of severe NAFLD compared to non-Hispanic Whites, while other Hispanics had a lower prevalence than Whites. Among all racial/ethnic groups, blacks had the lowest prevalence of NAFLD. These findings are consistent with other studies showing that Hispanics have a higher prevalence of NAFLD and blacks a lower prevalence of NAFLD compared to non-Hispanic Whites (2, 17).

The study found positive associations between moderate NAFLD and older age (50+ years old), high-risk waist-to-hip ratio, BMI (overweight and obese), and high serum triglyceride. Additionally, there were strong associations between severe NAFLD and age of 50–64, high waist-to-hip ratio, BMI, high-density lipoproteins, serum triglycerides, and HbA1c. The identified risk factors for NAFLD are consistent with other studies that found that an increased risk of NAFLD is associated with obesity, age, and other metabolic and cardiovascular diseases (13–15).

Importantly, the associations were modified when we stratified by sex. The increased odds of NAFLD observed for Mexican Americans and increased ALT levels was only present for males. Increases in odds observed for low levels of HDL and high levels of AST were observed only in females. When examining age-adjusted prevalence of NAFLD by stage, males had the highest prevalence of severe NAFLD followed by pre-menopausal women, followed by menopausal women without hormone therapy. Women on hormone therapy had a prevalence of severe NAFLD that was similar to that of males, although the sample size for this analysis was low. In the multivariable adjusted model, menopausal status did not affect the association between NAFLD and race/ethnicity, likely attributed to reduction in female hormones such as estrogen (29). Furthermore, the differences in risk factors related to gender suggests that the etiology or pathogenesis of NAFLD differ between the sexes. Previous studies had similarly found that in the adult population, men have a higher prevalence of NAFLD compared to women (22–42% in men vs. 13–24% in women) (15, 30, 31). In the Mexican pediatric population, NAFLD was more frequent in boys than girls (24.51% in boys vs. 11.96% in girls) (32). Previous studies based on NHANES III data found NAFLD is significantly more prevalent in men than in women (23, 33–38). Using NHANES 2017–2018 data, several studies saw that men had higher prevalence than women in both non-Hispanic white and Hispanic populations (39–41). Several factors may contribute to why we saw slightly different results, including that we did not combine Mexican Americans with other Hispanics; we characterized female sex by menopausal status; we were able to differentiate NAFLD from alcoholic fatty liver due to the availability of alcohol consumption data; and the CAP score cutoffs used to classify hepatic steatosis differed. Importantly, we also identified NAFLD related risk factors that differed by sex, which previous studies have not done. When examining reproductive-age groups, previous reports found that in the post-menopausal age, prevalence of NAFLD in men and women is comparable, whereas women in the premenopausal age have a lower prevalence compared to men (15, 30, 42, 43). The findings are consistent with our results, showing males had higher odds of NAFLD relative to non-menopausal but not menopausal females. There is evidence for gender differences in metabolic, inflammatory, and hormonal pathways that contribute to the differences observed in NAFLD (29–31). It will be important to investigate these differences further, with particular attention to differences by race/ethnicity.

### Strengths

First, the current study uses data from the most recently released 2017/2018 cycle of NHANES, making the analysis more relevant in determining current prevalence and risk factors. Second, the data are a large national representative sample of the non-institutionalized population in the US. Third, FibroScan^®^, the method used to detect hepatic steatosis in the 2017/2018 cycle is more sensitive than some of the other methods used such as liver enzymes or CT, although it is less sensitive than other methods like magnetic resonance spectroscopy (44). Fourth, the new data categorize Mexican Americans separately from Americans of other Hispanic background, revealing an important dissociation of the prevalence in Mexican Americans compared to other Hispanic populations.

### Limitations

One limitation of our study is that the NHANES data are cross-sectional, so we cannot determine the causal direction between NAFLD and its associated factors. Additionally, some variables, such as smoking, physical activity, menopausal status and hormone replacement were collected by self-report, so these estimates are prone to some recall bias. Numbers in the menopausal group on hormone replacement were low (*n* = 110), so a lack of finding in the AOR of NAFLD in menopausal women not taking hormone therapy compared to those taking hormone therapy may be due to limited power. Although we controlled for major confounders and robust associations, it is possible that other unknown confounders could account for the associations found.

### Conclusions

In conclusion, this study shows that the racial/ethnic difference in NAFLD differs by gender, with Mexican American men being at higher risk to develop the most severe form of the disease. The results indicate a gender disparity in NAFLD among the Hispanic US population. Our results also serve as another demonstration that the Hispanic American population is genetically and culturally diverse and care should be taken to avoid generalizing across groups of different backgrounds and gender. Screening and interventions that specifically target Mexican American males are needed to increase awareness about NAFLD and its prevention.

## Data Availability Statement

The datasets presented in this study can be found in online repositories. The datasets analyzed for this study can be found at the CDC https://www.cdc.gov/nchs/nhanes/index.htm.

## Ethics Statement

The studies involving human participants were reviewed and approved by NCHS of the CDC. The patients/participants provided their written informed consent to participate in this study.

## Author Contributions

MS, KS, DP, and DK performed analysis. MS, KS, DP, DK, and DE drafted the manuscript. MS, KS, DP, DK, VP, AZ, DE, SN, and TF reviewed and edited the manuscript. All authors contributed to the article and approved the submitted version.

## Funding

This study was supported by the National Institutes of Health (grants NIMHD R01MD012579, U54MD007598, S21MD000103, NIDA R24DA017298, and NCATS UL1TR000124).

## Conflict of Interest

The authors declare that the research was conducted in the absence of any commercial or financial relationships that could be construed as a potential conflict of interest.

## Publisher's Note

All claims expressed in this article are solely those of the authors and do not necessarily represent those of their affiliated organizations, or those of the publisher, the editors and the reviewers. Any product that may be evaluated in this article, or claim that may be made by its manufacturer, is not guaranteed or endorsed by the publisher.
